# Effect of *Salvia sclarea* L. extract on growth performance, antioxidant capacity, and immune function in lambs

**DOI:** 10.3389/fvets.2024.1367843

**Published:** 2024-04-10

**Authors:** Xiaoling Ma, Yujie Niu, Shanshan Nan, Wenju Zhang

**Affiliations:** College of Animal Science and Technology, Shihezi University, Shihezi, China

**Keywords:** *Salvia sclarea* L. extract, lambs, growth performance, immune function, antioxidant capacity

## Abstract

The aim of this experiment is to explore the effects of *salvia sclarea* extract on the growth performance, apparent nutrient digestibility, antioxidant capacity, and immune function of lambs. Sixty female lambs (Chinese Merino sheep) aged 2 months and weighing 20 ± 2 kg were selected and randomly divided into five groups of twelve lambs in each. While the control group (CK) received only basal feed, the experimental group was supplemented with different concentrations of *salvia sclarea* extract in the basal feed at 0.04 mL/kg (group CL1), 0.08 mL/kg (group CL2), 0.12 mL/kg (group CL3), and 0.16 mL/kg (group CL4). The feeding period was 85 days, including 15 days of pre-feeding and 70 days of regular feeding. Body weight and feed intake were recorded during the test period, and blood was collected at the end of the test for the determination of immune and antioxidant indices. The results showed that the average daily gain and average daily feed intake of lambs were significantly increased in CL3 group compared to CK group (*p* < 0.05). Also, the apparent nutrient digestibility of crude protein and neutral detergent fiber was significantly increased (*p* < 0.05). The Dry matter, acid detergent fiber and Ether extract were not significantly different (*p* > 0.05). The serum levels of superoxide dismutase, catalase, glutathione peroxidase, and antioxidant capacity were significantly higher in the CL2, CL3, and CL4 groups compared to CK group, while malondialdehyde levels were significantly lower (*p* < 0.05). The serum levels of immune globulin A, immune globulin G, immune globulin M, interferon-γ, and interleukin-10 were significantly higher and the levels of tumor necrosis factor-α and interleukin-1β were significantly lower in the CL2, CL3, and CL4 groups (*p* < 0.05). In conclusion, the addition of *salvia sclarea* extract to the ration promotes growth performance and nutrient digestion in lambs. Improvement of immune response by increasing immunoglobulin and cytokine concentrations. And it enhances the antioxidant status by increasing the antioxidant enzyme activity in lambs.

**Introduction:**

This study aimed to explore the effects of *Salvia sclarea* extract on the growth performance, apparent nutrient digestibility, antioxidant capacity, and immune function of the lambs.

**Methods:**

Sixty female lambs (Chinese Merino sheep) aged 2 months and weighing 20 ± 2 kg were selected and randomly divided into five groups of 12 lambs each. The control group (CK) received only basal feed, whereas the experimental group was supplemented with different concentrations of *salvia sclarea* extract in the basal feed at 0.04, 0.08, 0.12, and 0.16 mL/kg (CL1, CL2, CL3, and CL4, respectively). The feeding period was 85 days, including 15 days of pre-feeding and 70 days of regular feeding. Body weight and feed intake were recorded during the test period, and blood was collected at the end of the test to determine immune and antioxidant indices.

**Results:**

The results showed that the average daily weight gain and feed intake of the lambs were significantly higher in the CL3 group than in the CK group (*p* < 0.05). In addition, the apparent nutrient digestibility of crude protein and neutral detergent fiber increased significantly (*p* < 0.05). The dry matter, acid detergent fiber, and ether extract were not significantly different (*p* > 0.05). Serum levels of superoxide dismutase, catalase, and glutathione peroxidase and antioxidant capacity were significantly higher in the CL2, CL3, and CL4 groups than in the CK group, whereas malondialdehyde levels were significantly lower (*p* < 0.05). The serum levels of immune globulin immune globulin A, immune globulin G, immune globulin M, interferon-γ, and interleukin-10 were significantly higher and the levels of tumor necrosis factor-α and interleukin-1β were significantly lower in the CL2, CL3, and CL4 groups (*p* < 0.05).

**Discussion:**

In conclusion, the addition of the *S. sclarea* extract to the diet promoted growth performance and nutrient digestion in lambs. Immune response was improved by increasing Ig and cytokine concentrations. It enhances antioxidant status by increasing antioxidant enzyme activity in lambs.

## Introduction

Early postweaning stressors in lambs release glucocorticoids and coincide with a decrease in growth hormones, which suppress the immune system and reduce growth performance (1). A dietary change from milk to solid feed is considered a major stressor after weaning. The lack of a fully functional rumen in recently weaned ruminants reduces nutrient digestibility (2). Nutritional strategies have recently emerged. It has been proposed as a key factor in improving animal health and welfare as well as increasing livestock productivity (3). Dietary composition has long been recognized as an important factor in animal health, with significant effects on the acquired and innate immune systems, especially on inflammation (4). A number of compounds and products derived from plant-derived byproducts have been reported to show proinflammatory or anti-inflammatory effects and therapeutic responses and can trigger the expected response of the animal body to production parameters (5).

*Salvia sclarea* L. is a native Asian plant that is widely used in various fields including food, medicine, oils, and landscaping (6). It originated in Europe and is now primarily cultivated in France, Russia, and other countries. This plant was introduced into China in the early 1970s and cultivated in the Shaanxi and Henan provinces. In 2011, it was introduced into the Zhaosu area of Xinjiang, which has a cultivated area of approximately 1,000 ha per year. The plant produces 30 t of raw material and 60 kg of essential oil per ha (7). Previous phytochemical studies have identified and isolated various bioactive compounds, including flavonoids, volatile oils, fatty acids, triterpenes, and phenolic compounds, from *S. sclarea* frutescens (8). Owing to these biological components, pharmacological effects have been shown, including anti-inflammatory, antioxidant, and antibacterial effects (9). Previous studies have shown that dietary supplementation with *S. sclarea* improves meat quality without adversely affecting growth performance or carcass characteristics (10). However, few studies have investigated the effects of *S. sclarea* extract on lambs. Based on this, this study aimed to provide valuable scientific insights and powerful references for the rational application of *S. sclarea* extract in lamb production by adding different levels of *S. sclarea* extract to the growth performance, apparent nutrient digestibility, serum immunity, and antioxidant indices of lambs.

## Materials and methods

All experimental procedures were performed in strict accordance with guidelines and were reviewed and approved by the Institutional Animal Bioethics Committee of Shihezi University (Xinjiang, China).

### Experiment material

The *S. sclarea* extract (essential oil) used in the experiment appeared to be a light-yellow liquid. It was prepared by hydrodistillation of the flowers, leaves, and stems of *S. sclarea*. The active ingredients were linalyl acetate (54.79%) and linalool (30.22%), as detected using LC–MS. The purity of the *S. sclarea* extract was 85%. It was produced in the East Industrial Park of Zhaosu County, Xinjiang, China.

### Experimental design, animals, and management

Sixty female lambs (Chinese Merino sheep) aged 2 months and weighing 20 ± 2 kg were selected and randomly divided into five groups of 12 lambs each. The control group (CK) received basal feed alone, whereas the experimental group was supplemented with different gradients of *S. sclarea* extract in the basal feed at 0.04, 0.08, 0.12, and 0.16 mL/kg (CL1, CL2, CL3, and CL4, respectively). The feeding period was 85 days, including 15 days of pre-feeding and 70 days of normal feeding, and the sheep were fed daily at 06:00 h and 18:00 h. *Salvia sclarea* extract was precisely and uniformly mixed with the ration in strict accordance with the experimental design additive ratios. The sheep were housed in single-cage enclosures (1.5 × 1 × 1 m) with free access to water, and the sheds, water troughs, and troughs were cleaned and sterilized regularly to record daily feed intake and body weight. The composition and nutritional levels of the basal rations are listed in Table 1.

**Table 1:** Composition and nutrient levels of the basal diet (DM basis, %).

Ingredient	Content	Nutrition level^b^	Content
Corn	27.2	ME/(MJ/Kg)	12.74
Soybean meal	14.9	CP	15.43
NaCl	0.5	DM	42.11
Alfalfa Hay	21.5	NDF	33.75
Silage Corn	35.5	ADF	3.23
Premix^a^	0.4	Ca	0.28
Total	100	P	0.23

a Premix is provided per kilogram of feed. Each kilogram of trace element premix contains 5 mg of CuSO_4_·5H_2_O, 30 mg of FeSO_4_·7H_2_O, 20 mg of MnSO_4_·5H_2_O, 20 mg of ZnSO_4_·7H_2_O, 20 mg of KI, 40 mg of Na_2_SeO_3_, and 50 mg of CoCl·6H_2_O. VA 3000 IU, VD 400 IU, VE 90 IU.

b Metabolic energy is the calculated according to NRC (11), while other indicators are measured values.

### Sample collection and measurement methods

#### Growth performance

The lambs were weighed on the 1st and lower 70th days of the positive trial period and recorded as the initial body weight (IBW) and final body weight (FBW) of the test lambs, respectively, both on an empty stomach before the morning feeding. During the trial period, the amount of feed and leftovers were recorded in detail for each bureau pen per day, which was used as the basis for calculating the average daily feed intake (ADFI, ADFI = total feed intake/experimental days), average daily gain (ADG, ADFI = total feed intake/experimental days), and feed to weight ratio (F/G, F/G = ADFI/ADG) of the lambs.

### Nutrient apparent digestibility

Ration and fecal samples were collected during the last 3 days of the positive trial period. Samples were collected using the partial collection method, in which the ewes in each group were placed in homemade collection bags at 09:00 h and feces were recovered at 17:00 h each day. A sample of 200 g of feces was added to 10 mL of 10% sulfuric acid for nitrogen fixation and stored at −20°C for measurement. The collected grain and manure samples were placed in an oven at 65°C for 48 h, weighed after 24 h of moisture return, and prepared as analytical samples by crushing through an 80 mesh sieve. The dry matter (DM), crude protein (CP), crude ash (Ash), ether extract (EE), Ca, and P contents were determined using the methods of AOAC (2010) (12). The content of neutral and acid detergent fibers (NDF and ADF, respectively) was determined according to the method described by Van Soest et al. (13). The apparent digestibility of nutrients in lambs was determined using the acid-insoluble ash (AIA) endogenous indicator method (14).

### Serum biochemical and antioxidant indices

At the end of the experiment, 10 mL of blood was collected from the jugular vein of the sheep using a disposable syringe, and the serum was separated statically and stored frozen (−20°C) for subsequent experiments.

Serum immunity indicators, including antibodies: immunoglobulin G (IgG), immunoglobulin A (IgA) and immunoglobulin M (IgM), cytokines: tumor necrosis factor-α (TNF-α), interleukin-1β (IL-1β), interleukin-2 (IL-2), were measured using enzyme-linked immunosorbent assay (ELISA) method according to the instructions of the kit, Interleukin-4 (IL-4), Interleukin-6 (IL-6), Interleukin-8 (IL-8) and Interleukin-10 (IL-10) and antioxidant indexes: total antioxidant capacity (T-AOC), superoxide dismutase (SOD), glutathione peroxidase (GSH-Px), catalase (CAT), and malondialdehyde (MDA). The kits were purchased from Nanjing Jianjian Bioengineering Institute. Each sample was tested using an enzyme-labeling analyzer to detect the corresponding absorbance and a standard curve was established according to the manufacturer’s instructions to determine the concentration of the target factor.

### Statistical analysis

All data were analyzed using one-way ANOVA, linear analysis, and quadratic correlation using the SPSS software (version 22.0; SPSS, Inc., Chicago, IL, United States). Duncan’s method was used for multiple comparisons, and the experimental data were expressed as the mean and standard error of the mean (SEM). *p* < 0.05 indicated a significant difference, *p* < 0.01 indicated highly significant difference, and 0.05 ≤ *p* < 0.10 indicated a trend.

## Results

### Effect of *salvia sclarea* extract on the growth performance of lambs

No significant difference (*p* > 0.05) was observed in the initial weight of lambs in each group. The final weight of lambs in the CL4 group was significantly higher than that in the CK group (*p* < 0.05), and there was no significant difference (*p* > 0.05) among the CL2, CL3, and CL4 groups. Compared to the CK group, the ADG and ADFI of lambs in the CL3 and CL4 groups supplemented with *Salvia sclarea* extract were significantly higher, and the F/G was significantly lower (*p* < 0.05). The ADG of CL1, CL2, CL3, and CL4 increased by 7.97, 16.56, 23.31, and 34.35%, respectively, compared to that of the CK group (Table 2).

**Table 2:** Effect of *salvia sclarea* extract on the growth performance of lambs.

Items	Treatment					SEM	*p*-value		
	CK Group	CL1 Group	CL2 Group	CL3 Group	CL4 Group		Treat	Linear	Quadratic
Initial weight/kg	19.80	19.70	19.90	19.80	20.40	0.27	0.938	0.661	0.819
Final weight/kg	31.20^b^	32.05^ab^	33.29^ab^	33.94^ab^	35.78^a^	1.76	0.014	0.471	0.007
ADG/(g/d)	163.00^c^	176.00^c^	190.00^b^	201.00^ab^	219.00^a^	21.71	<0.01	<0.01	<0.01
ADFI/(kg/d)	1.27^b^	1.31^ab^	1.35^ab^	1.40^a^	1.44^a^	0.068	0.021	0.043	0.014
F/G	7.79^a^	7.44^ab^	7.10^b^	6.96^b^	6.58^c^	0.46	<0.01	<0.01	<0.01

The same superscript letters in peer data indicate no significant differences (*p* > 0.05), while different letters indicate significant differences (*p* < 0.05). CK, control group; CL1: 0.04 mL/kg; CL2: 0.08 mL/kg; CL3: 0.12 mL/kg; CL4: 0.16 mL/kg.

### Effect of *salvia sclarea* extract on the apparent digestibility of lambs

The apparent digestibility of DM, ADF, and EE in the four experimental groups CL1, CL2, CL3 and CL4 with the addition of *S. sclarea* extract was higher than that in the CK group, but the difference was not significant (*p* > 0.05). The apparent digestibility of NDF in the CL2 group was significantly higher than that in the CK group (*p* < 0.05); however, there was no significant difference among the CL1, CL3, and CL4 groups (*p* > 0.05). The apparent digestibility of CP and NDF in the CL3 group was significantly higher than that in the CK group (*p* < 0.05); however, there was no significant difference between the CL1 and CL3 groups (*p* > 0.05) (Table 3).

**Table 3:** Effect of *salvia sclarea* extract on apparent digestibility of lambs (%).

Items	Treatment					SEM	*p*-value		
	CK Group	CL1 Group	CL2 Group	CL3 Group	CL4 Group		Treat	Linear	Quadratic
DM	78.80	79.72	79.23	81.80	80.39	1.17	0.610	0.814	0.423
CP	70.20^b^	72.05^ab^	73.29^ab^	74.94^a^	73.78^ab^	1.80	0.048	0.313	0.029
NDF	63.34^b^	66.83^a^	66.97^a^	68.84^a^	65.81^ab^	2.01	0.043	0.102	0.241
ADF	51.27	53.42	54.13	54.49	53.67	1.25	0.647	0.428	0.460
EE	79.79	81.36	82.63	83.96	82.58	1.56	0.717	0.836	0.517

DM, dry matter; CP, crude protein; NDF, neutral detergent fibre; ADF, acid detergent fibre; EE, ether extract; CK, control group; CL1: 0.04 mL/kg; CL2: 0.08 mL/kg; CL3: 0.12 mL/kg; CL4: 0.16 mL/kg.

### Effect of *salvia sclarea* extract on serum antioxidant indices in lambs

**Figure 1:**
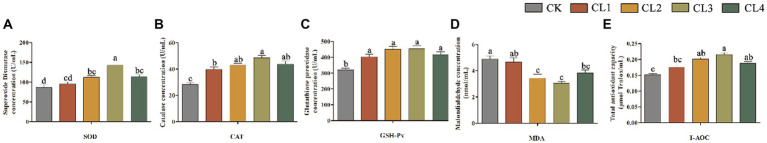
Effect of *salvia sclarea* extract on serum antioxidant indexes in lambs. **(A)** SOD; **(B)** CAT; **(C)** GSH-Px; **(D)** MDA; **(E)** T-AOC, CK, control group; CL1: 0.04 mL/kg; CL2: 0.08 mL/kg; CL3: 0.12 mL/kg; CL4: 0.16 mL/kg. Lower case letters in each bar chart indicate significant difference, the same letter indicates no significant difference (*p* > 0.05), and different lower case letters indicate significant difference (*p* < 0.05).

Compared with the CK group, the levels of SOD, CAT, GSH-Px, and T-AOC in the serum of lambs in the CL2, CL3, and CL4 groups treated with *S. sclarea* extract were significantly higher, whereas the MDA content was significantly lower (*p* < 0.05). Moreover, with an increase in the amount of *S. sclarea* extract, the content of SOD, CAT, GSH-Px, and T-AOC in the four experimental groups first increased and then decreased, whereas the MDA content showed the opposite trend. The CL1 group with 0.04 mL/kg *S. sclarea* extract had significantly higher CAT and GSH-Px content than the CK group (*p* < 0.05), whereas the content of MDA, SOD, and T-AOC was not significantly affected (*p* > 0.05). The SOD content was significantly higher (*p* < 0.05) in the CL3 group with the addition of 0.12 mL/kg *S. sclarea* extract than in the other four groups (Figure 1).

### Effect of *salvia sclarea* extract on serum immunity indices in lambs

**Figure 2:**
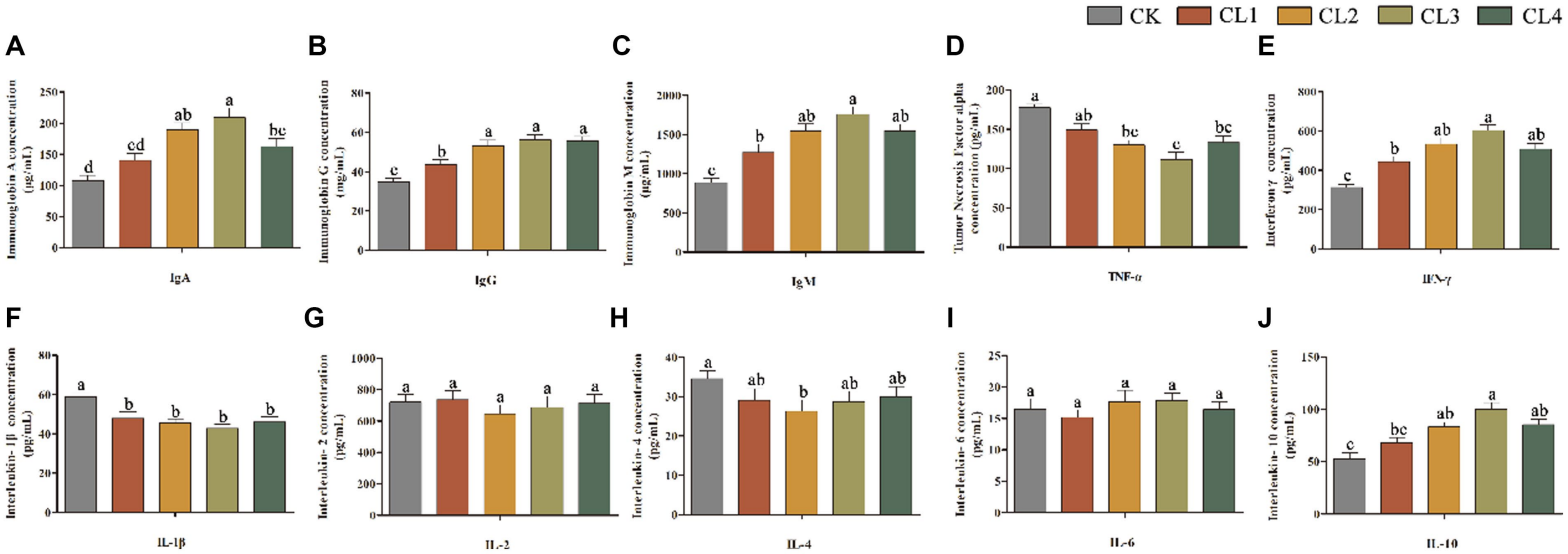
Effect of *salvia sclarea* extract on serum immunity indexes in lambs. **(A)** IgA; **(B)** IgG; **(C)** IgM; **(D)** TNF-α; **(E)** IFN-γ; **(F)** IL-1β; **(G)** IL-2; **(H)** IL-4; **(I)** IL-6; **(J)** IL-10, CK, control group; CL1: 0.04 mL/kg; CL2: 0.08 mL/kg; CL3: 0.12 mL/kg; CL4: 0.16 mL/kg. Lower case letters in each bar chart indicate significant difference, the same letter indicates no significant difference (*p* > 0.05), and different lower case letters indicate significant difference (*p* < 0.05).

Compared with the CK group, the content of IgA, IgG, IgM, IFN-γ, and IL-10 in the serum of lambs in the CL2, CL3, and CL4 groups was significantly higher (*p* < 0.05); that of TNF-α, and IL-1β was significantly lower (*p* < 0.05); and that of IL-2 and IL-6 showed no significant difference (*p* > 0.05). The IL-4 content decreased in the test groups CL1, CL2, CL3, and CL4 with the addition of *S. sclarea* extract; however, the difference between the CL2 and CK groups was significant (*p* < 0.05), whereas the rest were not significantly different (*p* > 0.05). The content of IgA, IgG, IgM, IFN-γ, and IL-10 in the serum of lambs in the CL3 group was higher than that in the other three test groups (Figure 2).

## Discussion

*Salvia sclarea* has long been grown in China as a medicinal and food plant, and has attracted attention for its unique active substances (9). Studies have shown that the aromatic *S. sclarea* is rich in phenolic acids (caffeic and rosemarinic acids), flavonoids, and volatiles (monoterpenes and sesquiterpenes) (15), among other active ingredients that enhance the immunity and antioxidant capacity of the animal and promote its growth and development (8). In this study, we found that the addition of *S. sclarea* frutescens extract to lamb rations had a positive effect on the growth performance of lambs by improving feed conversion efficiency and optimizing the nutrient utilization. Moreover, the feed intake of lambs increased with an increase in *S. sclarea* extract addition, and the feed intake significantly increased by 10.24% when the addition reached 0.12 mL/kg. This indicates that the unique aromatic odor due to the richness of terpenes, alkenes, and aromatic compounds improved the palatability of the feed and induced appetite in the lambs (16). Feed palatability can effectively increase the feed intake of lambs, increase their dietary intake, and improve their daily weight gain and feed utilization. Another study showed that phenolics in *S. sclarea* extracts could increase the activity of digestive enzymes by entering the intestinal tract (17). This reduces the number of harmful bacteria in the intestinal tract including *Escherichia coli*, *Staphylococcus aureus*, and *Bacillus subtilis* (18). Beneficial bacteria colonize the intestine, consume free oxygen, create a low-oxygen environment, and inhibit the growth of harmful aerobic bacteria and spoilage microorganisms. The joint action of the two reduces harmful bacteria on the consumption of nutrients to promote growth (19).

The maintenance of good production performance in animals is closely related to the digestion and metabolism of various nutrients in the body. Increasing nutrient digestibility can promote the absorption of nutrients such as DM and CP, thus promoting growth performance to a certain extent. The test group of lambs fed the *S. sclarea* extract had a higher apparent digestibility of CP and NDF than the CK group. No differences in the apparent digestibility of DM, EE, or NDF were found between the diets. In the present study, the concentrations of ingredients in the experimental TMR diet were similar. Therefore, these differences were attributed to the incorporation of the *S. sclarea* extract. There were some differences in the chemical compositions and bioactivities of the diets. The variable response of apparent digestibility in the diet treatment groups may be partly due to the higher CP of these lambs, as these factors positively influenced the degree of digestibility of the rations (20).

A positive correlation between oxidative stress and disease has been widely documented in animals. Antioxidant capacity reflects the body’s ability to scavenge free radicals accumulated in cells and tissues and protect the structure and function of cell membranes from damage by peroxides (21). MDA is a product of the lipid peroxidation reaction, and its content reflects the degree of lipid peroxidation in the body, which in turn reflects the degree of cellular attack by free radicals. The results of our study showed that the addition of *S. sclarea* extract to the ration increased serum CAT, SOD, GSH-Px activity, and T-AOC and decreased serum MDA content in lambs, with the best effect in the 0.12 mL/kg addition group. This indicated that the *S. sclarea* extract improved the performance and health of lambs by effectively reducing oxidative stress. Similar to the results of Deng et al. (22), the addition of *S. sclarea* to the diet of lambs significantly increased T-AOC activity and MDA content in the liver and improved the oxidative status of muscle and meat quality.

Li et al. (23) found that the dietary addition of *S. sclarea* extract increased serum SOD activity and decreased MDA content in the liver, spleen, and jejunal mucosa of piglets. The *S. sclarea* extract used in this study is rich in phenolic acids, flavonoids, and other bioactive components. Phenolic acids can combine with peroxyl radicals to reduce or eliminate free radicals. Phenolic hydroxyl groups can chelate transition metal ions to block biological oxidation (24). Terpenoids can increase the activity of antioxidant enzymes in animals and thus exert their antioxidant capacity (25). Flavonoids such as lignans, rosmarinic acid, and apigenin have powerful antioxidant properties (26). Based on this, we hypothesized that *S. sclarea* extract enhances serum CAT, SOD, and GSH-Px activities in lambs by competitively scavenging reactive oxygen species. Therefore, it has great potential as an antioxidant to prevent oxidative damage during livestock production.

Immunoglobulins are humoral immune effector molecules that play an important role in the immune system of young animals, and serum immunoglobulin levels can be used to evaluate the health status of an organism (27). IgA, IgM, and IgG, play a major role in the immune response. As a class of immunologically active molecules, they specifically bind to antigens and remove them via sedimentation and phagocytosis (28). They mediate immune and inflammatory responses when an animal is infested with bacteria and viruses and play an important role in the body’s immune response and immunoregulation (29). In this study, the *S. sclarea* extract affected the immune response in lambs by inducing IgG and IgM production. These responses protect lambs from pathogenic and nonpathogenic immune attacks. Linoleic and linolenic acids are “essential fatty acids” converted into eicosapentaenoic acid (EPA) and docosahexaenoic acid (DHA). Most studies have shown that EPA and DHA can clinically attenuate T cell immune-mediated inflammatory diseases. And α-linolenic acid enhances the immunity of the body (30). Previous studies demonstrated that essential fatty acids exert anti-inflammatory effects. When the animal body suffers from acute or chronic inflammation, as well as infection, it inhibits the production of prostaglandin 2 by secreting inflammatory factors and plays a role in regulating immune function (31). Liu et al. injected different doses of linolenic acid into the duodenum of dairy cows through a fistula and found that serum IgG levels increased significantly and prostaglandin 2 levels decreased in the 200 g/day dose group (32). Moreover, *S. sclarea* extract contains phenolics that increase leukocyte phagocytosis and can increase the release of IgA, IgM, and IgG. Regulating the expression of cytokines and increasing the expression of antibodies in the serum enhances the ability of the body to clear pathogens, thus strengthening its immune function (33).

As important components of cellular immunity, cytokines are critical for lymphocyte development and the subsequent functional activity of the peripheral immune system (34). Cytokines are produced by immune and non-immune cells and can be categorized into proinflammatory cytokines produced by a variety of immune cells (such as IL-1β, IL-6, and TNF-α) and anti-inflammatory cytokines (such as IL-4, IL-10) (35). Among them, IL-1β can induce cells to secrete inflammatory factors and inflammatory transmitters, triggering the body’s inflammatory response (36). IL-6 promotes immune cell differentiation and enhances their functional activity. IL-1β secretes IL-2 via T cells, whereas IL-6 induces IL-2 production by activating the NF-κB signaling pathway through signal transduction. IL-2 is a core substance in the immunoregulatory network of the body, reflecting the initiation of the immune response and playing an important regulatory role in both cellular and humoral immunity (37). IL-10 is an important anti-inflammatory factor secreted by Tregs and is regulated by the immunomodulatory factor IL-4, which can be used to determine the level of immunity in lambs (38). In this study, the addition of *S. sclarea* extract to the ration decreased the levels of proinflammatory cytokines IL-1β and TNF-α and increased the serum anti-inflammatory cytokine IL-10, thereby modulating the immune response. Lignans, a constituent of *S. sclarea* extract, inhibited the production of TNF-α in mouse serum (39). Moreover, linolenic acid, an anti-inflammatory agent in a mouse model of colonic inflammation, had a protective effect against TNBS-induced colitis through the Th1/Th2/Th17 pathway with inflammation-reducing efficacy (40). In addition, a previous study demonstrated that dietary supplementation with phenolic compounds inhibited LPS-induced expression of proinflammatory cytokines IL-1β and IFN-γ through NF-κB and MAPK signaling pathways (41). These results demonstrate the potential of *S. sclarea* extracts to reduce the infection load and inflammatory responses *in vivo*.

## Conclusion

In conclusion, the addition of 0.12 mL/kg *S. sclarea* extract to the diet improves the growth performance of lambs by increasing feed intake and nutrient digestibility. It also improves the health status of lambs by increasing their serum antioxidant capacity and immune function. *S. sclarea* extract has great potential as a feed additive in livestock production. It is also worth exploring its potential use in various animal production environments.

## Data availability statement

The original contributions presented in the study are included in the article/supplementary material, further inquiries can be directed to the corresponding author.

## Ethics statement

The animal studies were approved by the Institutional Animal Bioethics Committee of Shihezi University. The studies were conducted in accordance with the local legislation and institutional requirements. Written informed consent was obtained from the owners for the participation of their animals in this study.

## Author contributions

XM: Writing – original draft. YN: Writing – review & editing. SN: Writing – review & editing. WZ: Writing – review & editing.
